# Machine learning-based identification of a consensus immune-derived gene signature to improve head and neck squamous cell carcinoma therapy and outcome

**DOI:** 10.3389/fphar.2024.1341346

**Published:** 2024-04-10

**Authors:** Xueying Hu, Haiqun Dong, Wen Qin, Ying Bin, Wenhua Huang, Min Kang, Rensheng Wang

**Affiliations:** ^1^ Department of Radiation Oncology, The First Affiliated Hospital of Guangxi Medical University, Nanning, Guangxi, China; ^2^ Key Laboratory of Early Prevention and Treatment for Regional High Frequency Tumor (Guangxi Medical University), Ministry of Education, Nanning, Guangxi, China; ^3^ Guangxi Key Laboratory of Immunology and Metabolism for Liver Diseases, Nanning, Guangxi, China

**Keywords:** biomarker, immunotherapy, prognosis, head and neck squamous cell carcinoma, a machine learning, consensus immune-derived gene signature

## Abstract

**Background:**

Head and neck squamous cell carcinoma (HNSCC), an extremely aggressive tumor, is often associated with poor outcomes. The standard anatomy-based tumor–node–metastasis staging system does not satisfy the requirements for screening treatment-sensitive patients. Thus, an ideal biomarker leading to precise screening and treatment of HNSCC is urgently needed.

**Methods:**

Ten machine learning algorithms—Lasso, Ridge, stepwise Cox, CoxBoost, elastic network (Enet), partial least squares regression for Cox (plsRcox), random survival forest (RSF), generalized boosted regression modelling (GBM), supervised principal components (SuperPC), and survival support vector machine (survival-SVM)—as well as 85 algorithm combinations were applied to construct and identify a consensus immune-derived gene signature (CIDGS).

**Results:**

Based on the expression profiles of three cohorts comprising 719 patients with HNSCC, we identified 236 consensus prognostic genes, which were then filtered into a CIDGS, using the 10 machine learning algorithms and 85 algorithm combinations. The results of a study involving a training cohort, two testing cohorts, and a meta-cohort consistently demonstrated that CIDGS was capable of accurately predicting prognoses for HNSCC. Incorporation of several core clinical features and 51 previously reported signatures, enhanced the predictive capacity of the CIDGS to a level which was markedly superior to that of other signatures. Notably, patients with low CIDGS displayed fewer genomic alterations and higher immune cell infiltrate levels, as well as increased sensitivity to immunotherapy and other therapeutic agents, in addition to receiving better prognoses. The survival times of HNSCC patients with high CIDGS, in particular, were shorter. Moreover, CIDGS enabled accurate stratification of the response to immunotherapy and prognoses for bladder cancer. Niclosamide and ruxolitinib showed potential as therapeutic agents in HNSCC patients with high CIDGS.

**Conclusion:**

CIDGS may be used for stratifying risks as well as for predicting the outcome of patients with HNSCC in a clinical setting.

## Introduction

Head and neck squamous cell carcinoma (HNSCC) is the eighth most prevalent carcinoma worldwide (Sung et al., 2021). Given its unique anatomical location and lack of available screening strategies for early detection, HNSCCs are mostly unresectable or found early metastasis at diagnosis (Bonartsev et al., 2022). Treatment approaches for HNSCC are typically multimodal and multidisciplinary and include surgical resection, radiation, or chemotherapy combined with radiation (Johnson et al., 2020). Cetuximab is approved for use in either combination therapy or as monotherapy for unresectable HNSCCs (Vincenzi et al., 2010). Notably, the overall 5-year survival rate of HNSCCs has risen from 55% to 66% (Pulte and Brenner, 2010); however, overall treatment outcomes remain unsatisfactory. More recently, immunotherapy has come to be considered as an effective therapeutic option for most solid tumors (Billan et al., 2020). Since the first results of clinical trials aimed at immune checkpoint inhibitors (ICIs) were published in 2016 (Leemans et al., 2018), treatment of HNSCC has shifted to pembrolizumab or nivolumab, which are recommended as first-line treatments for locally advanced, recurrent, or metastatic HNSCCs (Ferris et al., 2016; Seiwert et al., 2016; Burtness et al., 2019). However, only 10%–20% of patients respond to these ICIs (Ferris et al., 2016; Seiwert et al., 2016). Thus, better clinical management as well as therapeutic approaches able to overcome the limitations of current targeted therapies and improve HNSCC prognosis are urgently needed.

The tumor–node–metastasis staging system is widely used to evaluate the clinical stage of cancers. However, HNSCCs are remarkably inter- and intra-tumorally heterogeneous owing to their complex anatomical structure, diverse etiologies, and inherent molecular changes that drive carcinogenesis. Hence, this traditional staging system is largely ineffective against HNSCCs and prevents clinicians from providing optimal treatment for patients with HNSCC, thereby leading to latent over-or-under treatment. The development of high-throughput sequencing has enabled molecular biomarkers, such as *EGFR*, *TP53*, *CDKN2A*, *CCND1*, and *PTEN*, to be identified as druggable genes (Leemans et al., 2018). However, small-molecule inhibitors have been effective only against some HNSCCs. Moreover, the use of mRNA or non-coding RNA expression in specific pathways (*e.g.*, metabolic reprogramming, epigenetic modification, and immunity) as molecular biomarkers remains scant. In addition, the application of multigene signatures in clinical practice is affected by important limitations, such as a dearth of appropriate modeling methods and a lack of strict validation via large multicenter cohorts. Therefore, identification of reliable molecular biomarkers that may help optimize drug therapy aimed at HNSCC remains crucial. To overcome these issues, 85 machine-learning algorithm combinations were used to assess the clinical value of mRNAs in HNSCCs and to construct a consensus immune-derived gene signature (CIDGS). This CIDGS was tested on 722 patients with HNSCC from several independent datasets. This study is expected to provide a new basis for predicting the outcomes and enhancing the treatment decision process for HNSCCs.

## Methods

### Review of datasets

The expression profiles, clinical characteristics and follow-up information pertaining to HNSCCs were acquired from The Cancer Genome Atlas (TCGA) and Gene Expression Omnibus (GEO) datasets. We enrolled 719 patients from three cohorts: TCGA-HNSC (*n* = 519), GSE41613 (*n* = 97), and GSE42743 (*n* = 103). Additionally, the IMvigor210 cohort (*n* = 298) and the GSE78220 cohort (*n* = 28) were incorporated into the validation analysis (Hugo et al., 2016; Mariathasan et al., 2018). The detailed information of five included cohorts were summarized in Supplementary Table S1. RNA-sequencing data from the TCGA-HNSC, IMvigor210, and GSE78220 cohorts were transformed using log-2 transformation. All data from the Gene Expression Omnibus database were obtained using the GeneChip Human Genome U133 Plus 2.0 Array (Affymetrix, Santa Clara, CA, United States). The collected data were preprocessed by the robust multiarray averaging (RMA) algorithm implemented in the “*affy*” package (Irizarry et al., 2003).

### Immune infiltration and consensus clustering estimation

The “*GSVA*” function (Charoentong et al., 2017) was applied for single-sample gene set enrichment analysis (ssGSEA) as well as for detecting the levels and biologic functions of infiltration immune cells in the training cohort (TCGA-HNSC). Based on the infiltration levels of immune cells in tumor samples and immune functions, resulting from ssGSEA, the “*ConsensusCluster*” package was used to perform a resampling-based method termed consensus clustering via *K*-means algorithms in order to investigate immune patterns (Wilkerson and Hayes, 2010). Moreover, the cumulative distribution function curves were synthetically constructed to determine the best cluster that could be utilized to divide into “immune-cold” and “immune-hot” categories (Senbabaoglu et al., 2014). Eight algorithms—CIBERSORT-abs, CIBERSORT, quanTIseq, ESTIMATE, MCPcounter, TIMER, EPIC, and xCELL—were employed to determine the robustness and authenticity of ssGSEA and consensus clustering results.

### Weighted correlation network analysis (WGCNA)

The co-expressed gene networks in the TCGA-HNSC cohort were constructed using the “*WGCNA*” function (Langfelder and Horvath, 2008). The standard for a scale-free network was defined by the best threshold *β*. A dynamic tree-cutting process was used to determine mRNA profiles and explore the correlation between immune them and clusters. The mRNA module with the most correlation was used for the follow-up analysis.

### Machine learning-derived CIDGS

To construct a promising CIDGS, gene expression profiles were converted into *z*-scores to strengthen comparability among different samples (Liu et al., 2022a; Xu et al., 2022). The procedure which was followed to generate signatures was as follows:

(1) Kaplan-Meier analysis was applied to identify prognostic genes in the TCGA-HNSC cohort.
(2) Ten machine learning algorithms—supervised principal components (SuperPC), Lasso, partial least squares regression for Cox (plsRcox), Ridge, stepwise Cox, random survival forest (RSF), survival support vector machine (survival-SVM), CoxBoost, elastic network (Enet), and generalized boosted regression modelling (GBM)—were applied. Briefly, high-throughput calculations were performed to determine the suitable signature. An RSF algorithm was performed using the “*randomForestSRC*” package. In particular, all pairs (ntree and mtry), which are pivotal RSF parameters, were defined as the number of trees in the forest and variables for splitting at each node, respectively. Moreover, a 10-fold cross-validation was used for a grid search on all pairs. The parameter with the top 1 *C*-index was recommended as the best pair. Three algorithms (Enet, Ridge, and Lasso) were conducted using the “*glmnet*” package. A Lambda algorithm was identified and the threshold of the L1–L2 trade-off parameter (*α*) was defined as 0–1 (interval = 0.1). The “*survival*” package was used to create the stepwise Cox model. Stepwise was applied according to the Akaike information criterion. Component-wise likelihood-based boosting was employed to fit a Cox regression model using the “*CoxBoost*” function. The “*optimCoxBoostPenalty*” function was employed to identify the optimal penalty. The “*cv.CoxBoost*” function was used to identify other tuning parameters. The CoxBoost function was used as the principal routine to determine the dimensions of the multivariate Cox model. The “*plsRcox*” function was used to fit a partial least squares regression generalized linear model. The number of components was identified by the “*cv.plsRcox*” function. The SuperPC model was conducted using the “*SUPERPC*” function and applied to generate a linear combination of variables or factors of interest, which captured the directions of the most variable factor in all datasets. The optimal threshold in SuperPC was assessed using the “*superpc.cv*” package according to a 10-fold cross-validation. The “*pre-validation*” function was applied to prevent issues arising from fitting of multivariate Cox regression models to other cohorts. The “*gbm*” package was used to evaluate the fit of the generalized boosted regression model. The “*cv.gbm*” package was used to determine an index for number trees. The survival-SVM model was applied using the “*survivalsvm*” function. The regression approach was incorporated into the censoring concept to determine the constraints on inequality in the support vector problem.
(3) A total of 85 algorithm combinations were applied to all included cohorts. The best signature was defined as that with the highest *C*-index.

### Retrieving known signatures of HNSCC

Fifty-one reported signatures were obtained from PubMed for analysis (Supplementary Table S2). Given that the cohorts lacked data on non-coding RNA these signatures were not included. The mRNA signature was constructed using ten machine learning algorithms as well as 85 algorithm combinations. Receiver operating characteristic (ROC) curve analysis and *C*-index calculation were performed to assess the clinical application value of the CIDGS.

### Genomic alteration landscape

Raw data on somatic mutations and the Human Methylation450 array were obtained from TCGA-HNSC datasets. The tumor mutation burden (TMB) was identified by calculating non-silent somatic mutations. To evaluate differences in genetic mutations, the “*maftools*” and “*ComplexHeatmap*” functions were applied to identify the 20 genes whose mutation frequencies were the highest. The association between the CIDGS and TMB was assessed via Spearman’s correlation analysis. Genomic Identification of Significant Targets in Cancer 2.0 (Mermel et al., 2011) was used to process data on copy number variation.

### Enrichment analysis

Potential molecular mechanisms were predicted via Gene set enrichment analysis (GSEA). Following differential analysis, all genes were ranked based on their log2 fold-change (log2FC). Using the “clusterProfiler” function, Gene ontology (GO) categories and Kyoto Encyclopedia of Genes and Genomes (KEGG) enrichment analysis were performed to determine potential biological roles as well as functional pathways. The top 10 biologic pathways were selected for visualization.

### Immune landscape

The ESTIMATE algorithm was used to estimate tissue components. The correlations between these parameters and the CIDGS score were evaluated using Pearson’s correlation coefficients. Next, the levels of immune cell populations were determined using CIBERSORT. Then, ssGSEA was used to assess the ability of the two CIDGS groups to resist tumor infiltration. Additionally, seven algorithms were employed to explore the association between the levels of immune cell populations and the CIDGS score. The levels of immune checkpoints in the two groups were analyzed. The association between immune checkpoints and the CIDGS score was further analyzed.

### Response to immunotherapy

Expression similarity between the two CIDGS groups and those who did not respond to ICIs were evaluated via subclass mapping (submap) analysis (Hoshida et al., 2007). Next, we evaluated the efficacy of immunotherapy in the two CIDGS groups. The IMvigor210 cohort (Mariathasan et al., 2018), which included patients with bladder cancer receiving atezolizumab, was analyzed to further verify the capability of the CIDGS score for predicting responsiveness to immunotherapy. Furthermore, the GSE78220 cohort (Hugo et al., 2016), which comprised melanoma patients treated with ICIs, was also included in the study.

### Prediction of potential drugs

The Genomics of Drug Sensitivity in Cancer was reviewed to assess drug responses and potential drug candidates (Yang et al., 2013). Potential small molecule inhibitors suitable for use in the high-CIDGS group were explored based on a previously published protocol (Yang et al., 2021). Sensitivity data pertaining to therapeutic agents aimed at cancer cell lines were acquired from two datasets [Cancer Therapeutics Response Portal (CTRP) and profiling relative inhibition simultaneously in mixtures (PRISM)], whereas gene expression profiles were acquired from the Cancer Cell Line Encyclopedia database. The area under the ROC curve (AUC) values were presented by two datasets (CTRP and PRISM) with higher AUC values indicating greater resistant to agents. Differential analysis of the treatment response was calculated via the Wilcoxon rank-sum test, with a log2FC > 0.2 threshold, indicating potential compounds corresponding to patients with high CIDGS. Furthermore, agents with AUC values that were negatively correlated with CIDGS scores were screened using Spearman’s correlation analysis (threshold *R* < −0.4). Thus, potential agents suitable for patients with high CIDGS scores were identified via intersection of the above analysis results.

The NCI-60 Human Tumor Cell Lines Screen database, which contains information on 60 different tumor cells from nine cancer types, was explored to assess the relationship of CIDGS-related genes and agent sensitivity, and Pearson correlation analysis via the “*ggplot2*” package with the CellMiner interface was applied to assess such correlations.

### Statistical analysis

R software (v4.1.3) was used to conduct all statistical analyses. Correlation matrices were determined via Pearson’s and Spearman’s correlation analyses. R package “*survminer*” was used to identify the optimal cutoff value. The “survival” function was applied to conduct Cox regression and Kaplan-Meier analyses, while the “*CompareC*” function was used to calculate the *C*-index. ROC curves were plotted using the “*pROC*” package. All analyses were based on two-tailed tests and statistical significance was set at *p* < 0.05.

## Results

### Construction of immune infiltration consensus clusters

**FIGURE 1:**
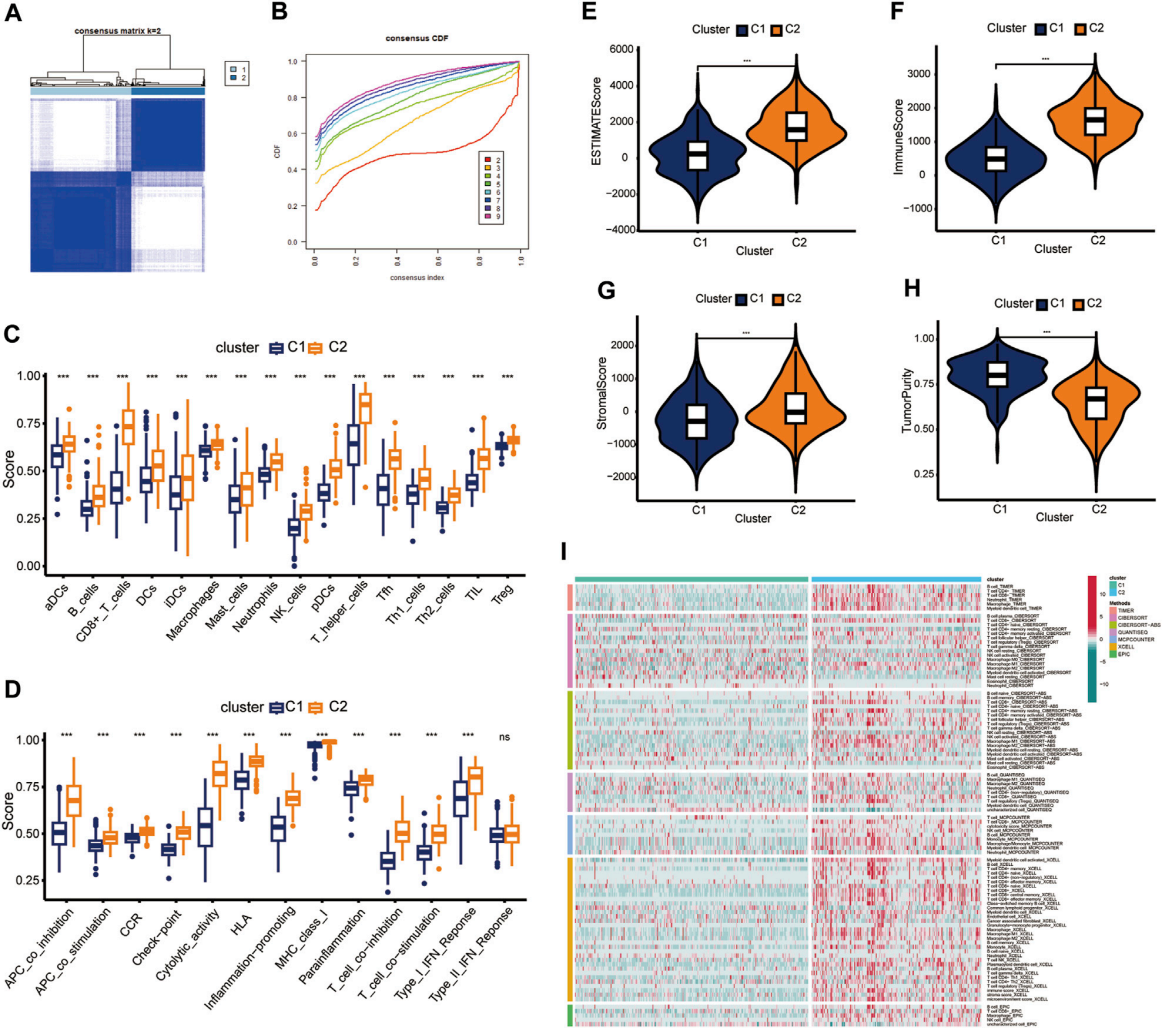
Identification of consensus clusters. **(A)** *k* = 2 was considered as the optimal number. **(B)** Cumulative distribution function (CDF) plots of the consensus indexes of different *k* values (by different indicators). **(C,D)** Immune cell populations and functions were verified by ssGSEA. **(E–H)** Differences in tumor components between clusters C1 and cluster C2. **(I)** Immune cell populations within C1 and C2 were assessed using different analysis tools. ns, not significant; ***p < 0.001.

Consensus cluster analysis was conducted using the ssGSEA algorithm (Wilkerson and Hayes, 2010; Charoentong et al., 2017), with TCGA-HNSC samples being divided into *k* (*k* = 2–9) clusters (Supplementary Figure S1). The results revealed that *k* = 2 was optimal (Figures 1A, B). C1 and C2 clusters differed significantly in relation to immune cell infiltration and biological function. Compared with the consensus cluster C1, C2 had significantly higher immune infiltration levels (Figures 1C, D). In addition, C2 showed markedly higher immune, stromal, and estimated scores (Figures 1E–G, *p* < 0.001), whereas cluster C1 showed higher tumor purity scores (Figure 1H, *p* < 0.001). Therefore, C1 and C2 were considered as representing “immune-cold” and “immune-hot” tumors, respectively. Other immune-related algorithms further verified that the ssGSEA results were stable and robust (Figure 1I).

### Identification of hub modules

**FIGURE 2:**
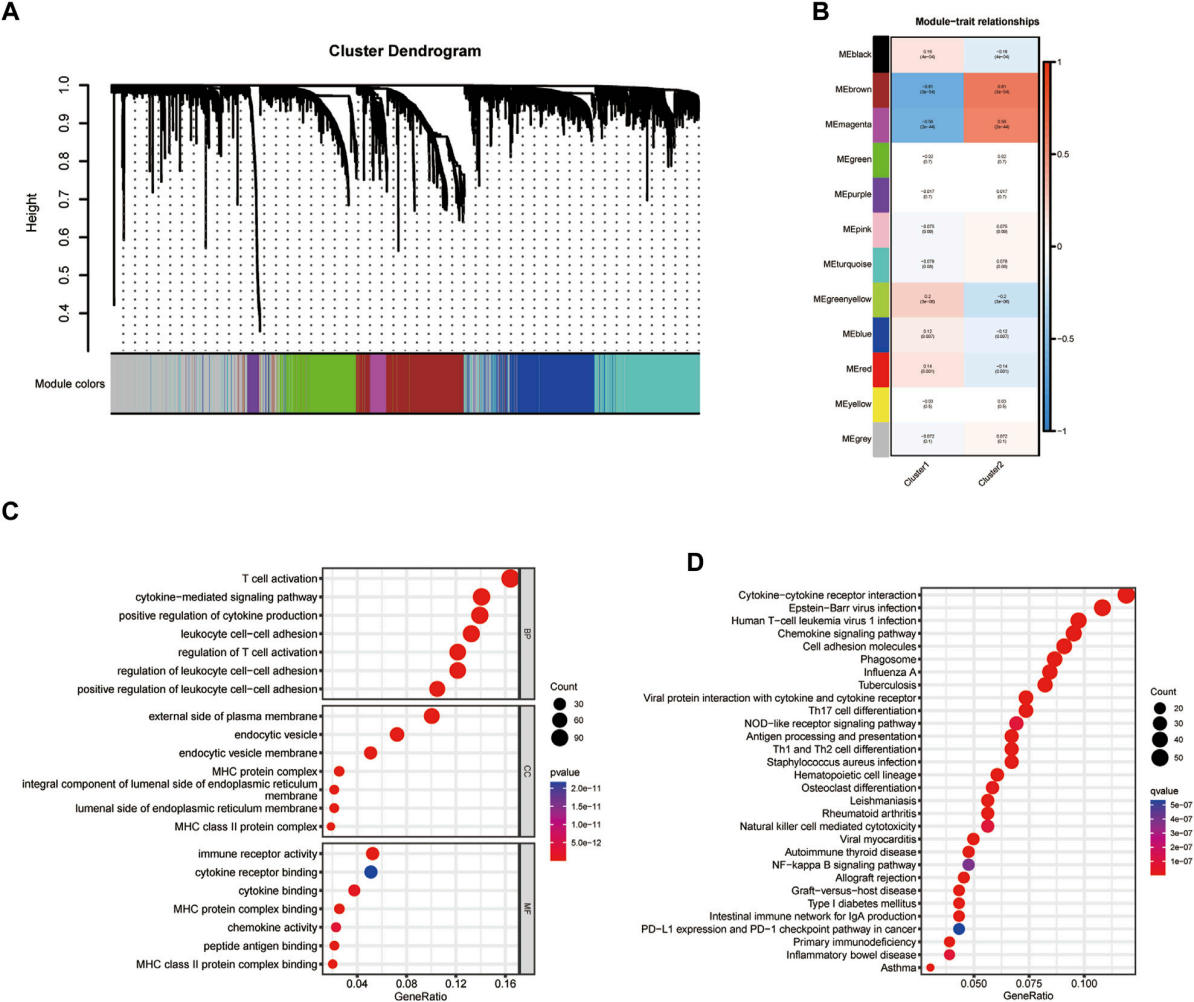
Identification of hub modules. **(A,B)** Weighted correlation network analysis (WGCNA) for cluster dendrogram design. **(C,D)** Gene ontology (GO) and Kyoto Encyclopedia of Genes and Genomes (KEGG) analyses for identifying hub modules.

Based on the cluster dendrogram, WGCNA was conducted to identify the genes most associated with immune infiltration (Figure 2A). The threshold of *β* was set to five (*R* ^*2*^ = 0.9; Supplementary Figure S2). We identified a total of 33 hub modules. Interestingly, the dark turquoise and blue modules, which comprised 763 genes, showed the highest correlation coefficients among all hub modules with immune infiltration features (Figure 2C). Gene ontology analysis revealed that all hub genes were markedly associated with several immune functions including “positive regulation of leukocyte cell-cell adhesion,” “cytokine-mediated signaling pathway,” “T cell activation,” “regulation of T cell activation,” “positive regulation of cytokine production,” and “regulation of leukocyte cell-cell adhesion” (Figure 2D). For cellular component, these genes were significantly found in the “endocytic vesicle,” “endocytic vesicle membrane,” “MHC class II protein complex,” “MHC protein complex,” and “integral component of luminal side of endoplasmic reticulum membrane” (Figure 2D). With respect to their molecular function, these immune-related genes were significantly related to “cytokine binding,” “chemokine activity,” “peptide antigen binding,” “cytokine receptor binding,” and “immune receptor activity” (Figure 2D). Moreover, KEGG analysis indicated that these genes were associated with several immune-related pathways, such as “phagosome,” “cytokine-cytokine receptor interaction,” “chemokine signaling pathway,” and “human T-cell leukemia virus one infection” (Figure 2E). These results revealed that the identified module genes correlated with immune-related pathways, demonstrating their involvement in immune regulation.

### Integrative construction of the CIDGS

**FIGURE 3:**
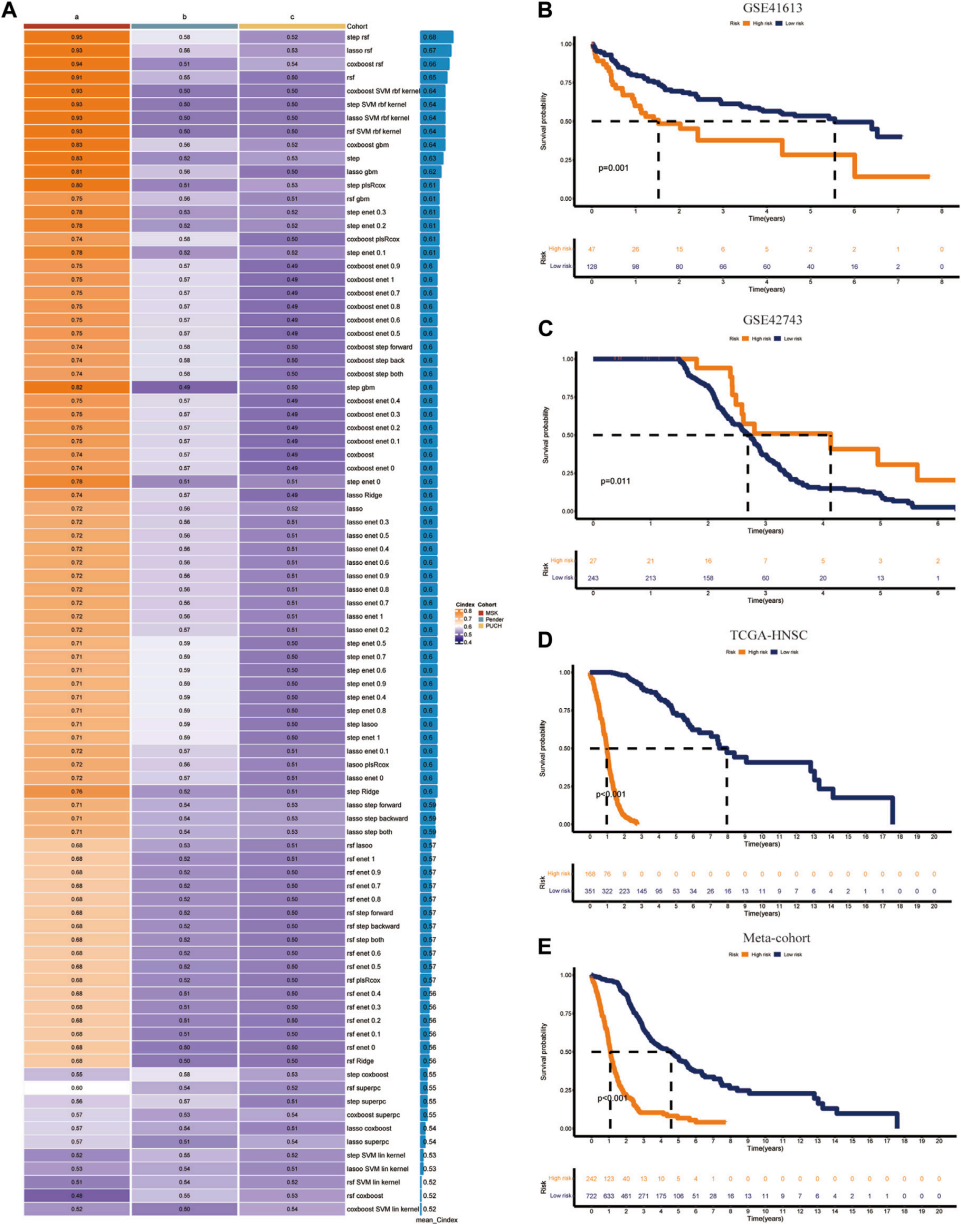
Integrative construction of CIDGS. **(A)** Eighty-five algorithm combinations were used to identify the CIDGS. The average *C*-index of three cohorts (TCGA-HNSC, GSE41613, and GSE42743) was calculated. **(B–E)** The overall survival in TCGA-HNSC, GSE41613, and GSE42743, and Meta-cohort datasets was analyzed using Kaplan–Meier curves.

Univariate Cox analysis identified 90 of the 236 identified immune-related genes as prognostic genes (Supplementary Table S3). These 90 prognostic genes were then used in machine learning algorithms to determine the CIDGS. In the TCGA-HNSC datasets, a 10-fold cross-validation framework was applied to fit 85 types of predictive model signatures via the LOOCV framework (Figure 3A). The *C*-index of each predictive signature across two validation cohorts was calculated (Figure 3A). Notably, the results indicated that a combination of RSF and stepwise Cox (direction = both), with an optimal C-index was 0.68, were the optimal predictive models (Figure 3A). Thus, 90 optimal immune-related genes were identified using this model. Next, the expression levels of these 90 optimal immune-related genes were weighted using regression coefficients to calculate the CIDGS score for each patient (Supplementary Table S4). Based on the highest cutoff value, HNSCCs were classified as high- or low-CIDGS groups. Kaplan-Meier analysis of the training datasets (TCGA-HNSC) confirmed that overall survival (OS) in the low CIDGS group (*p* < 0.05; Figure 3B), as well as in two other validation datasets (GSE41613 and GSE42743, *p* < 0.05; Figures 3C, D) and Meta-cohort datasets (*p* < 0.001; Figure 3E) was significantly higher. These results revealed that the CIDGS was highly stable and that it may be extrapolated across multiple independent cohorts.

### Comparative performance of CIDGS

**FIGURE 4:**
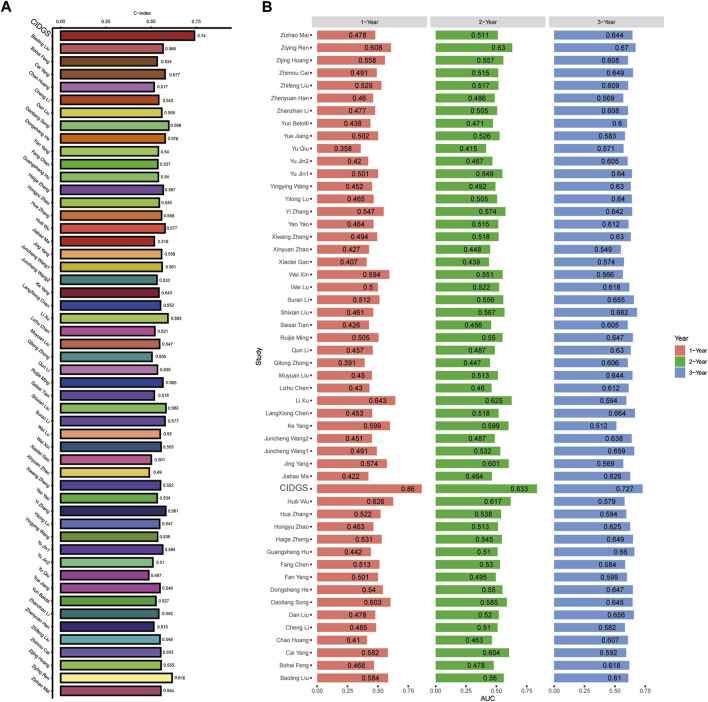
Comparison of CIDGS with previously reported signatures. **(A)** The average *C*-index of the CIDGS score and 51 HNSCC-related signatures. **(B)** ROC analysis of the CIDGS score and previously reported HNSCC-related signatures for predicting 1-, 2-, and 3-year prognoses.

Machine learning algorithms (Ahluwalia et al., 2021) were used to compare the performance of the CIDGS with that of 51 previously reported HNSCC-related signatures (Supplementary Table S5), as well as to identify predictive and prognostic gene signatures. A total of 51 mRNA signatures, which were related to several biological processes, including pyroptosis, immune checkpoints, glycolysis, autophagy, aging, inflammation, hypoxia, cytoproptosis, ferroptosis, lipid, N6-methyladenosine, and other hotspot mechanisms/processes, were comprehensively identified and included in the analysis. Assessment of the average *C*-index of all signatures indicated that the CIDGS had the highest *C*-index (Figure 4A). ROC curve analysis was used to assess the performance of the CIDGS in the TCGA-HNSC cohort. The AUCs for 1-, 2-, and 3-year overall survival were 0.86, 0.833, and 0.727, respectively (Figure 4B), further indicating that the prediction performance of the CIDGS, obtained via a combination of multiple machine learning algorithms, greatly outperformed those of other reported signatures.

### Nomogram construction

**FIGURE 5:**
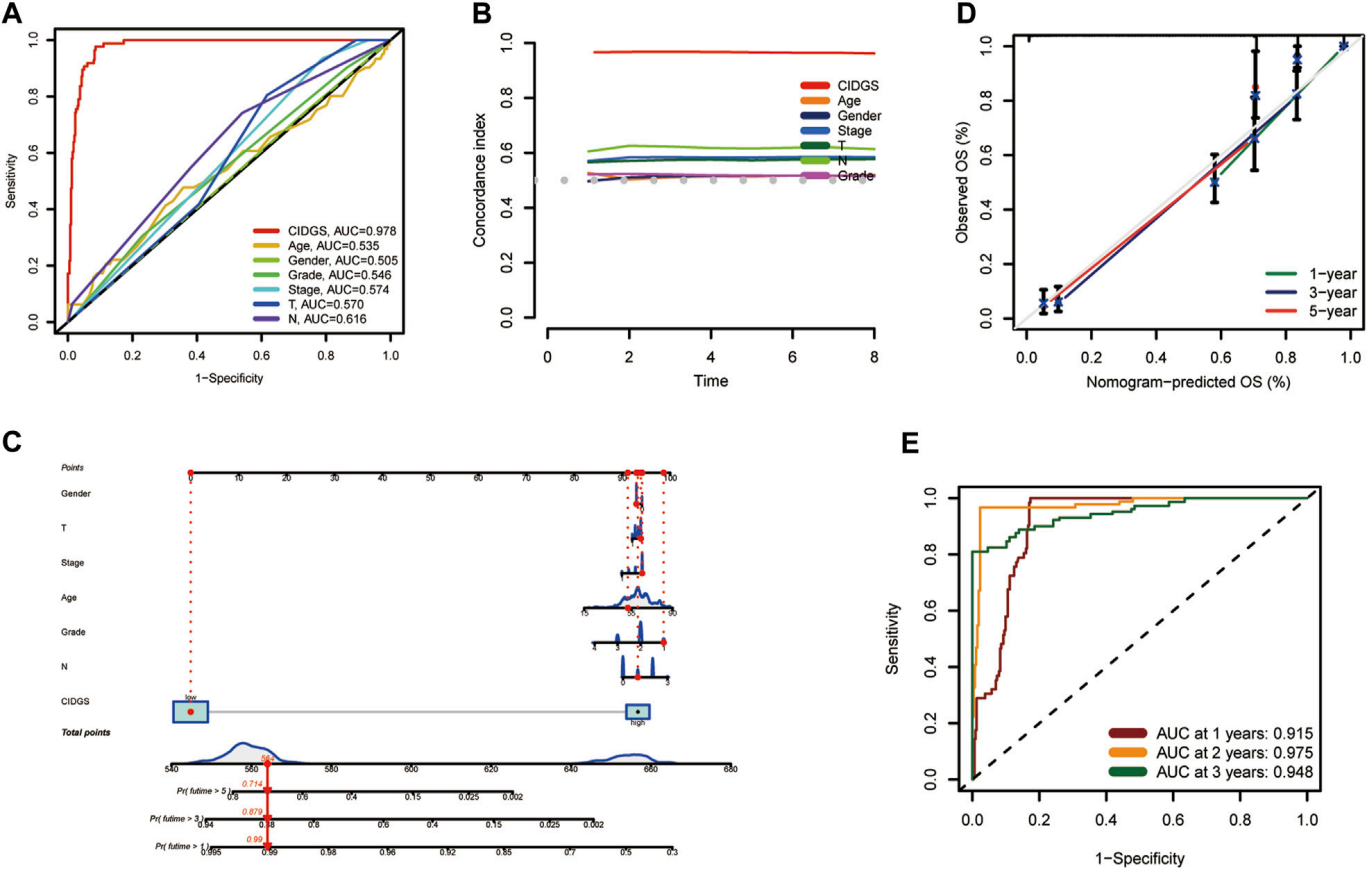
A prognostic nomogram. **(A)** ROC analysis of the CIDGS score and clinically relevant pathological factors. **(B)** *C*-index of the CIDGS and clinical factors for evaluating treatment outcome. **(C)** Nomogram model presenting the CIDGS and clinicopathological factors. **(D)** Nomogram model predicting overall survival using calibration curves. **(E)** Time-ROC analysis for predicting prognosis for the Meta-cohort. ***p < 0.001.

To further assess the stability and robustness of the CIDGS, its prognostic performance was compared with that of several clinical features. ROC analysis indicated that the CIDGS risk score and nomogram yielded an AUC which was significantly higher than those of the other clinical features in the TCGA-HNSC cohort (Figure 5A). Moreover, the CIDGS had the highest *C*-index, thereby demonstrating its solid and robust capability for evaluating the prognoses for HNSCCs (Figure 5B). Notably, time-dependent ROC analysis also indicated that the AUCs for 1-, 2-, and 3-year OS of the meta-cohort were 0.915, 0.975, and 0.948, respectively (Figure 5C). Therefore, a prognostic nomogram combining several clinical factors and the CIDGS score was constructed (Figure 5D). Furthermore, the calibration plot indicated that this prognostic nomogram effectively predicted actual survival outcomes (Figure 5E). Overall, the CIDGS showed an excellent predictive performance, thereby demonstrating its potential as a clinically relevant prognostic tool.

### Comprehensive analysis of CIDGS-related genes

**FIGURE 6:**
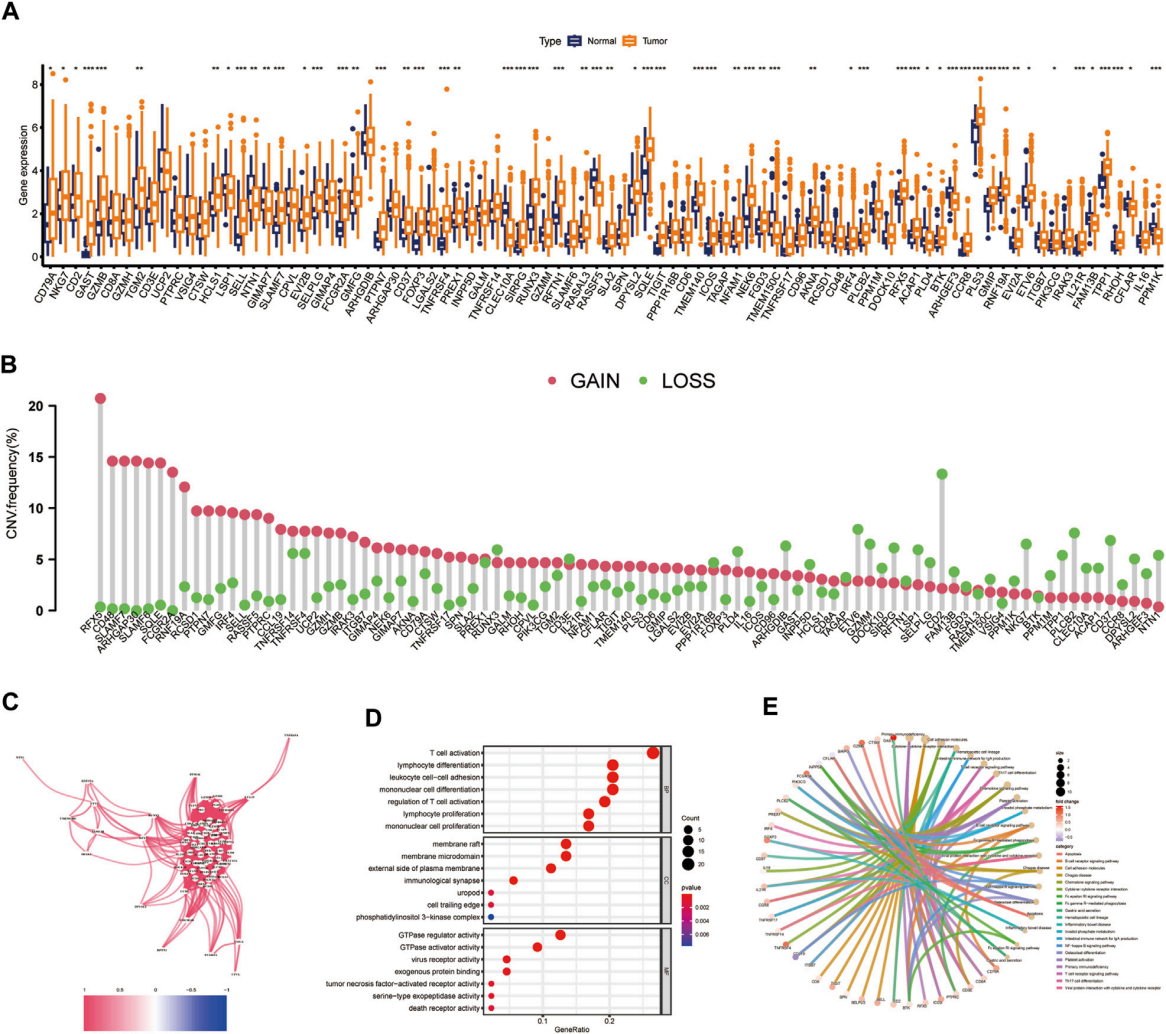
Landscape of CIDGS-related genes. **(A)** Expressions of CIDGS-related genes between HNSCC and healthy samples. **(B)** Frequencies of copy number variations (CNV) in CIDGS-related genes. **(C)** Correlation intensity between CIDGS-related genes. The correlation coefficient was set at 0.6. **(D,E)** GO and KEGG analyses of CIDGS-associated genes. ***p < 0.001; **p < 0.01; *p < 0.05.

The expression levels of 90 CIDGS-related genes in HNSCC tumors and healthy samples were determined. Interestingly, most CIDGS-related genes, such as *XRCC6*, *ST13*, *PES1*, *EFHD2*, *APOL2*, *ACTR2*, and *CDCP1*, were markedly upregulated in HNSCC tumor samples, whereas several other genes, such as *MARCO*, *MRC1*, and *CD83*, were upregulated in healthy samples (Figure 6A). Notably, the copy number variation mutation frequency varied among the CIDGS-related genes (Figure 6B). Additionally, the co-expression network of the CIDGS-related genes (with a cutoff value of 0.5) revealed a very high correlation strength between the genes (Figure 6C). GO analysis indicated that the CIDGS-related genes were mostly associated with several biologic functions, including “T cell activation,” “regulation of T cell activation,” “lymphocyte differentiation,” and “mononuclear cell differentiation,” among others (Figure 6D). In addition, KEGG analysis revealed that CIDGS-related genes were highly associated with several pathways, including “the NF-κB signaling pathway,” “the B cell receptor signaling pathway,” “the chemokine signaling pathway,” “cell adhesion molecules,” “the T cell receptor signaling pathway” (Figure 6E). These results indicated the latent tumorigenic function of CIDGS-related genes in HNSCCs.

### Characteristics of the immune landscape

**FIGURE 7:**
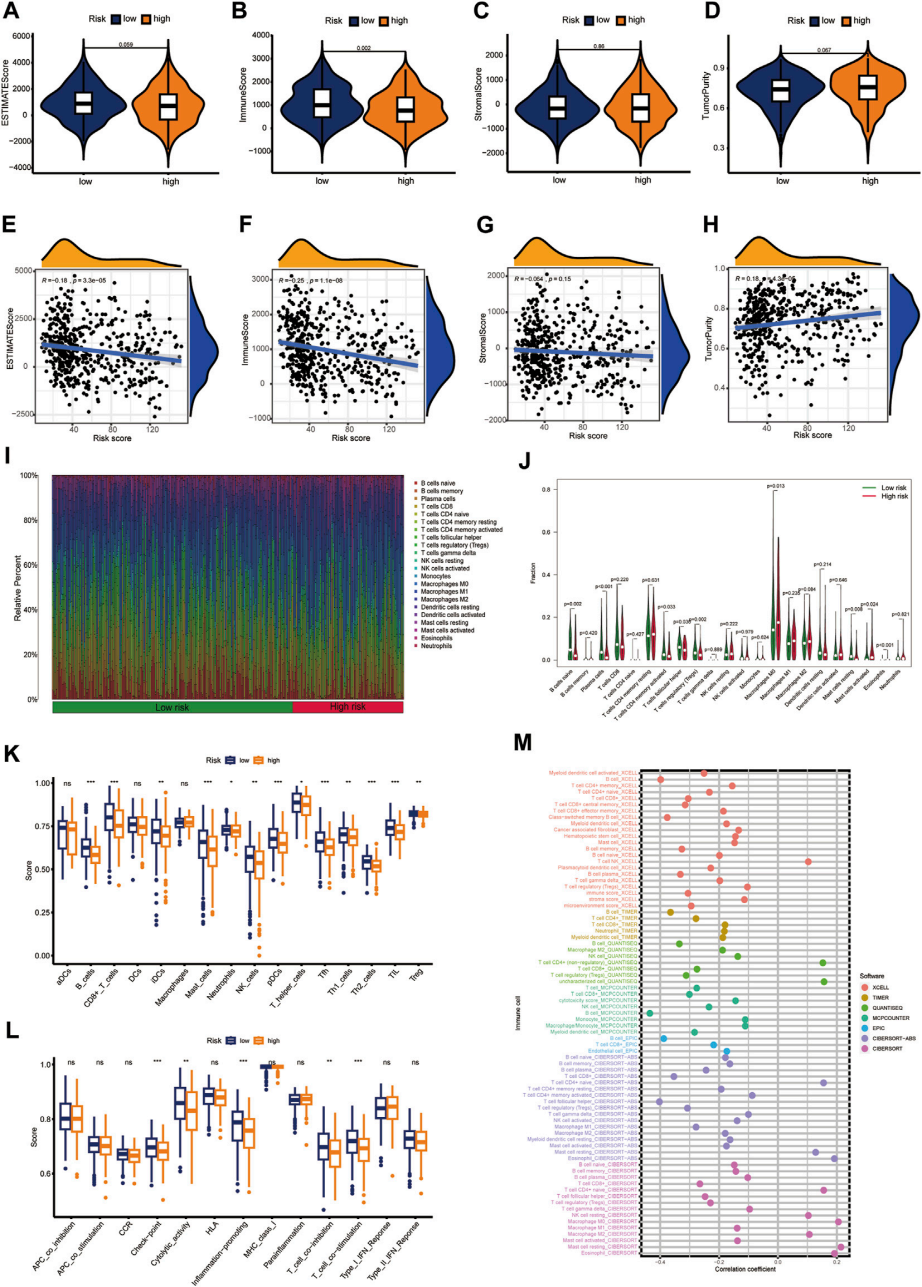
Characterization of the immune landscape. **(A–D)** Tissue components were assessed using the ESTMATE algorithm. **(E–H)** Associations between CIDGS score and tissue components. **(I,J)** The CIBERSORT algorithm was used to determine the proportion of immune cells. **(K,L)** Immune cell populations and functions were determined using ssGSEA. **(M)** Multiple algorithms were applied to assess the relationship between CIDGS and immune cell subtypes. ns, not significant; ***p < 0.001; **p < 0.01; *p < 0.05.

Multiple immune algorithms were used to explore immune landscapes. The immune scores in the low-CIDGS group were significantly higher (*p* = 0.002; Figure 7A), while its stromal and estimate scores showed a tendency to increase (*p* > 0.05; Figures 7B, C). Furthermore, patients with low CIDGS showed a tendency towards increased tumor purity (*p* > 0.05; Figure 7D). Notably, tumor purity and the CIDGS score were positively correlated, whereas the immune and estimate scores, but not the stromal score, were negatively correlated (Figures 7E–H). Additionally, an analysis of the immune landscape using the CIBERSORT algorithm indicated that the relative percentage of immune cells in patients with low CIDGS was remarkably higher (Figure 7I), indicating that the immune infiltration level was negatively associated with the CIDGS score. The ratio of the immune cell population in both groups was also examined (Figure 7J). The fractions of T follicular helper (*p* = 0.03), activated CD4 memory T (*p* = 0.033), naïve B (*p* = 0.002), plasma (*p* < 0.001), and T regulatory (*p* = 0.002) cells in patients with high CIDGS were remarkably lower, whereas those of activated mast cells (*p* = 0.024), eosinophils (*p* < 0.001), and M0 macrophages (*p* = 0.013) in patients with low CIDGS were significantly lower. Notably, the ssGSEA algorithm indicated that the infiltrations by natural killer, B, CD8^+^ T, interstitial dendritic, plasmacytoid dendritic, T follicular helper, T helper 1/2, and regulatory T cells, as well as tumor-infiltrating lymphocytes and neutrophils in patients with low CIDGS were obviously higher (*p* < 0.05; Figure 7K). Additionally, the checkpoint, T cell co-inhibition/stimulation, promotion of inflammation, and cytolytic activity in patients with low CIDGS were also remarkably upregulated (*p* < 0.05; Figure 7L). Correlation analysis using seven algorithms indicated that the levels of immune cell populations were negatively associated with the CIDGS score (Figure 7M). Thus, a low CIDGS score correlates with a greater immune infiltration level, and thus may help predict the immunotherapy effect.

### CIDGS predictive potential for response to immunotherapy

**FIGURE 8:**
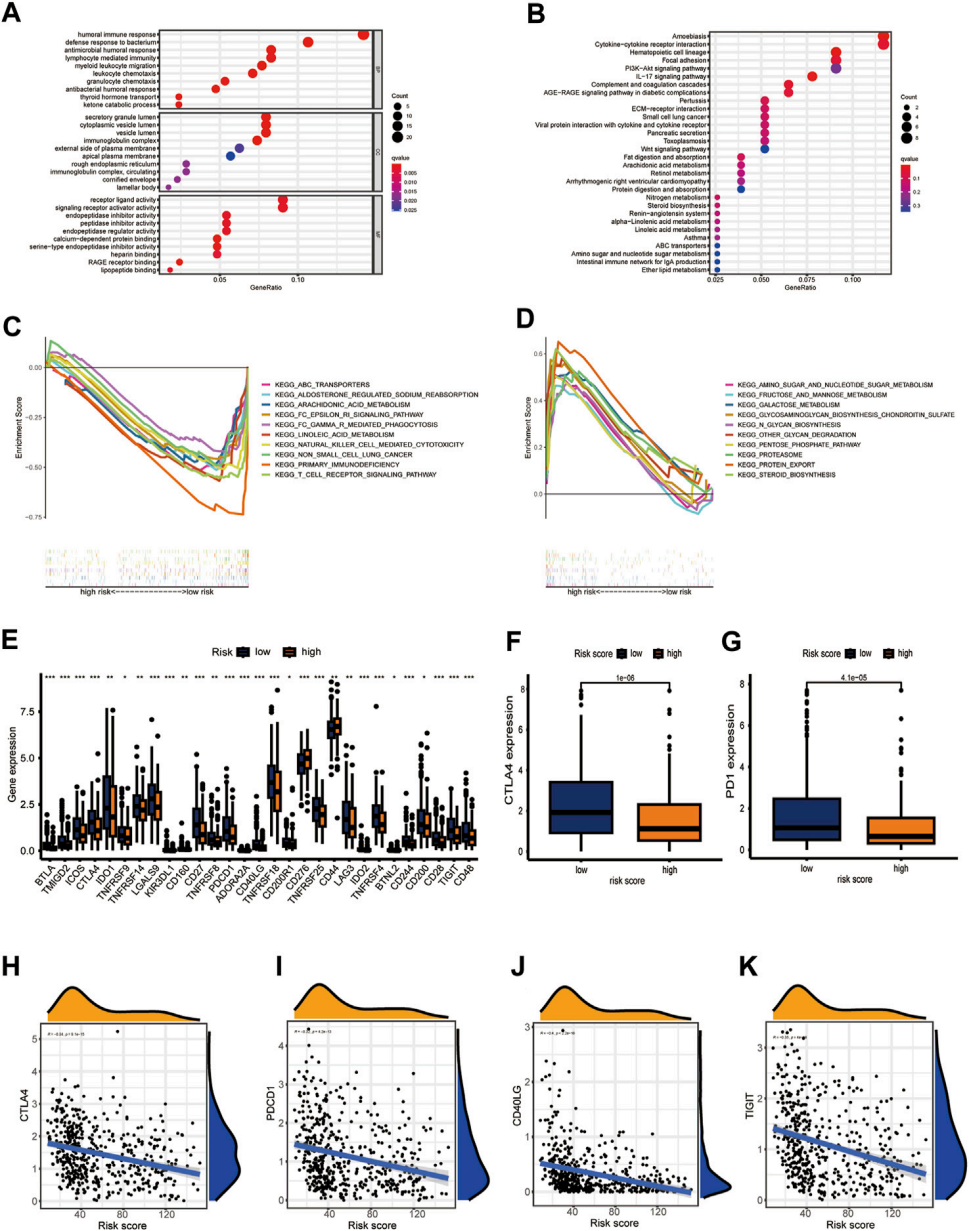
CIDGS potential for predicting response to immunotherapy. **(A,B)** GO and KEGG analyses of 90 CIDGS-associated genes. **(C,D)** GSEA based on the KEGG analysis. **(E)** Expression of immune checkpoint genes. **(F,G)** Expression of *CTLA4* and *PD-1*. **(H–K)** Correlation between CIDGS scores and *CTLA4*, *PDCD1*, *CD40LG*, and *TIGIT* levels. ***p < 0.001; **p < 0.01; *p < 0.05.

The above shown significant differences between the immune characteristics of the two CIDGS groups indicated that immune characteristics of CIDGSs that are developed using immune-related patterns may differ. Therefore, we predicted that sensitivity to immunotherapy would be different between HNSCCs with high and low CIDGS scores (Supplementary Table S6). GO enrichment analysis showed that “humoral immune responses,” “lymphocyte-mediated immunity,” “myeloid leukocyte migration,” and “leukocyte chemotaxis” were remarkably related to low CIDGS (Figure 8A). KEGG analysis further demonstrated that “the Wnt signaling pathway,” “the IL-17 signaling pathway,” “cytokine-cytokine receptor interactions,” “hematopoietic cell lineage,” and “the PI3K−Akt signaling pathway” were significantly associated with low CIDGS (Figure 8B). GSEA based on KEGG data of the two CIDGS groups was conducted to select the top 10 pathways. Several pathways, such as “T cell receptor signaling pathways,” “FC gamma-mediated phagocytosis,” “transporters,” and “natural killer cell-mediated cytotoxicity,” were found in the low-CIDGS group (Figure 8C), while “biosynthesis-related pathways” (*e.g.*, glycosaminoglycan, glycan, and steroid biosynthesis) and “metabolism-related pathways” (*e.g.*, amino and nucleotide sugars, fructose and mannose, and galactose metabolism) were associated with high CIDGS (Figure 8D). To better assess the immunotherapy response, we further explored the expression levels of 47 immune checkpoint members, including the TNF superfamily and B7CD28 family (Figure 8E). These results indicated that a high CIDGS score was associated with lower expression of *BTLA*, *TMIGD2*, *ICOS*, *CTLA-4*, *IDO1*, *LGALS9, CD27*, *TNFRSF8*, *PDCD1*, *CD40LG*, *TNFRSF18*, *CD200R1*, *TNFRSF25*, *LAG3*, *TNFRSF4*, *CD244*, *CD200*, *CD28*, *TIGIT*, and *CD48*, and higher expression of *CD276* and *CD44* (*p* < 0.05; Figure 8E). Interestingly, *CTLA4* and *PD-1* were highly expressed in patients with low CIDGS (*p* < 0.001; Figures 8F, G). Moreover, the expression levels of *CTLA4* (*R* = −0.34), *PDCD1* (*R* = −0.32), *CD40LG* (*R* = −0.4), and *TIGIT* (*R* = −0.2) were negatively associated with the CIDGS score (Figures 8H–K). These results revealed that HNSCCs with low CIDGS scores would benefit from treatment with immune checkpoint inhibitors and that several immune molecules showed potential as promising targets for immunotherapy.

### Validation of the CIDGS for immunotherapy

**FIGURE 9:**
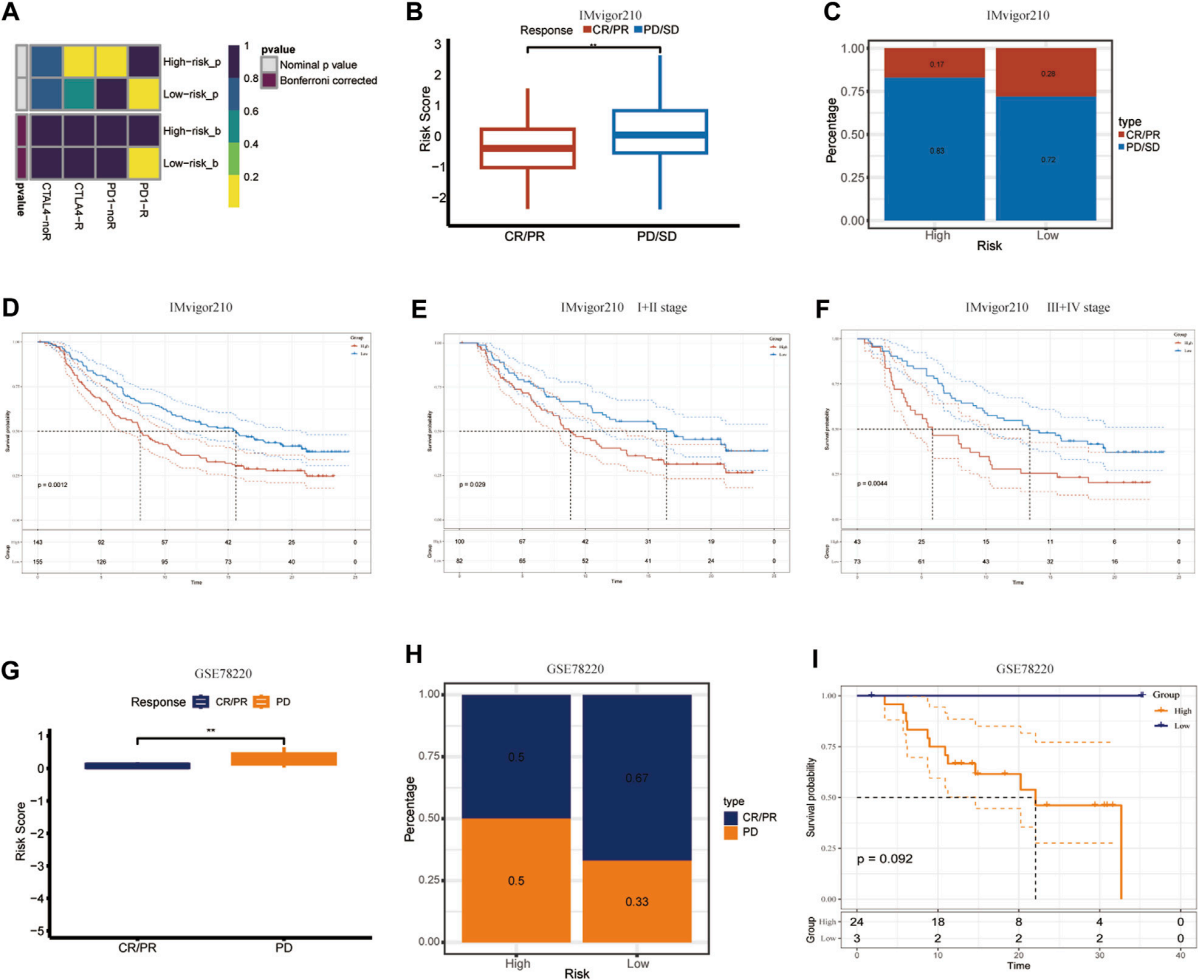
Validation of CIDGS to define PD-L1 blockade immunotherapy application. **(A)** Subclass mapping analysis in 47 patients pre-treated with immunotherapy. Smaller *p*-values represent higher similarity among paired expression profiles. **(B,C)** Differences in CIDGS scores among immunotherapy response groups and distribution of immunotherapy responses among two CIDGS groups in the IMvigor210 cohort. **(D–F)** Overall survival among two CIDGS groups in all patients, early stage patients, and in advanced patients from the IMvigor210 cohort. **(G–I)** CIDGS scores among immunotherapy response groups, immunotherapy responses among two CIDGS groups, and overall survival among two CIDGS groups in the GSE78220 cohort. **p < 0.01.

PD-1/PDL-1 immunotherapy is an anticancer treatment with synergistic survival benefits (Curran et al., 2010). Given that the low-CIDGS group shows higher expression levels of immune-related molecules and an activated tumor microenvironment (TME), we surmised that this group may be more responsive to immunotherapy. Thus, immune response testing algorithms, such as submap, were applied to assess the performance of CIDGS for predicting response to immunotherapy. Overall, patients with low CIDGS benefited from the superior efficacy of anti-CTLA-4 immunotherapy (Figure 9A). The prognostic performance of the CIDGS in immunotherapy was further verified in the IMvigor210 cohort, in which the response to immunotherapy agents was divided into four subgroups as follows: progressive disease (PD), stable disease (SD), partial response (PR), and complete response (CR). The CIDGS score of the CR/PR group was obviously lower than that of the SD/PD group (Figure 9B). Additionally, the percentage of CR/PR was distinctly higher in patients with low CIDGS (Figure 9C). Furthermore, patients with low CIDGS scores had remarkably longer OSs (*p* = 0.0012; Figure 9D). Significantly longer OS was also observed in stage I + II patients with bladder carcinoma (*p* = 0.029; Figure 9E), as well as in stage III + IV patients (*p* = 0.0044; Figure 9F). The GSE78220 cohort showed similar results (Figures 9G–I). These findings revealed that patients with low CIDGS may benefit from immune checkpoint inhibitors.

### Somatic mutational landscape and biological mechanisms

**FIGURE 10:**
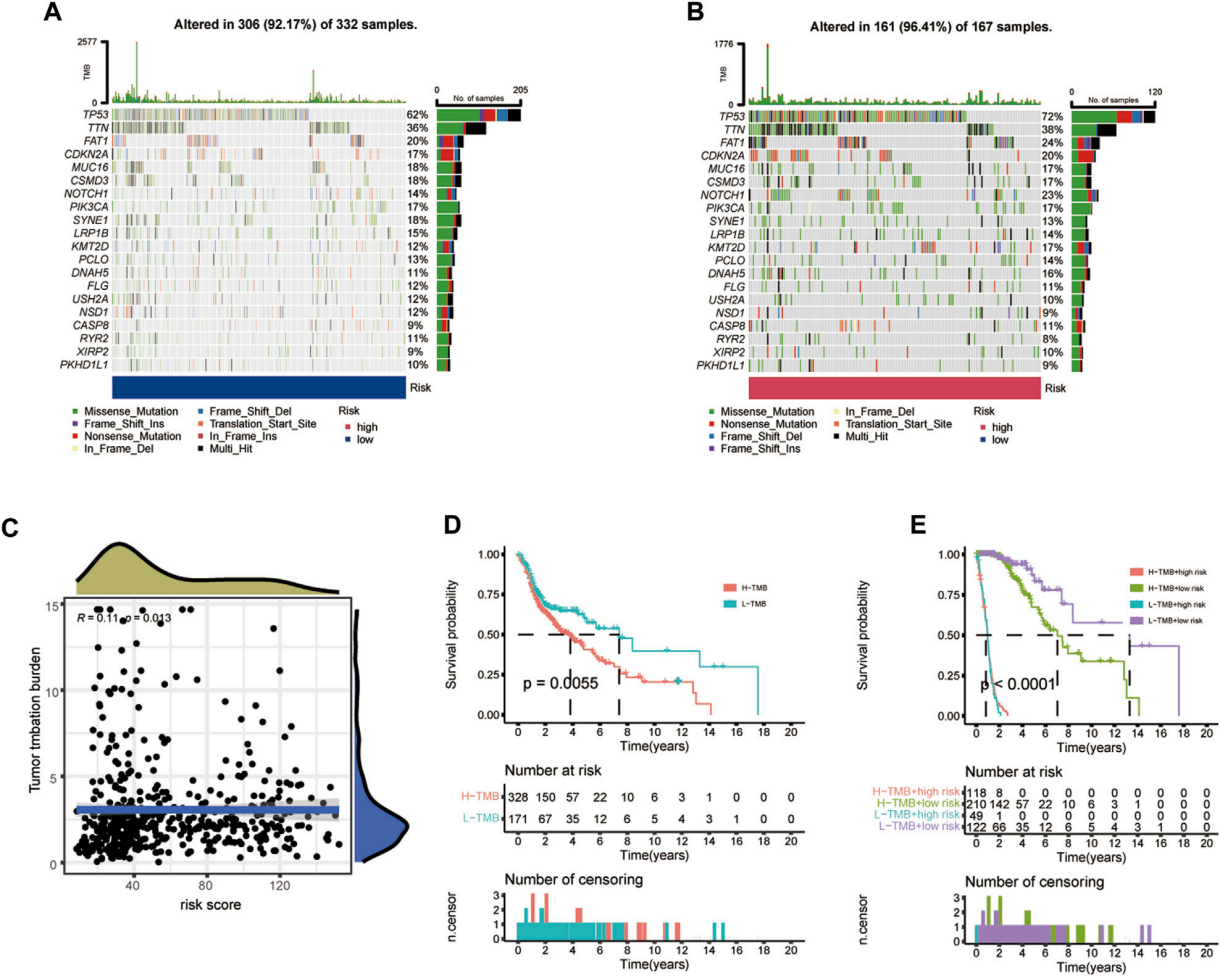
Correlation between CIDGS and TMB. **(A,B)** Mutation frequencies in two CIDGS groups. **(C)** Association between CIDGS and TMB. **(D)** Different overall survival in two TMB groups. **(E)** Overall survival in the different TMB groups combined with different CIDGS score groups.

The TMB, which is also considered as a molecular marker, was applied to calculate the number of somatic mutations (Merino et al., 2020). The differences between somatic mutations were further assessed. The waterfall plot indicated that the mutation rate was 92.17% (mutations in 306 of 332 samples) in the low- CIDGS group and 96.41% (mutations in 161 of 167 samples) in the high-CIDGS group, respectively (Figures 10A, B). Among these, frequently mutated genes, including *TP53*, *TTN*, *FAT1*, *CDKN2A*, *NOTCH1*, *KMT2D*, and *DNAH5*, in the high-CIDGS group exhibited high mutational frequency. Spearman’s analysis revealed that the TMB score was positively associated with the CIDGS score (*R* = 0.11, *p* = 0.013; Figure 10C). Based on the optimal cutoff value (median TMB score), HNSCCs were classified as high- and low-TMB groups. Kaplan-Meier analysis indicated that the low-TMB group was associated with a significantly longer OS (*p* = 0.0055; Figure 10D). We further explored whether the TMB or the CIDGS score would be the better survival predictor for patients with HNSCC. Unexpectedly, patients with low TMB/low CIDGS conferred the greatest benefits to overall survival (*p* < 0.0001; Figure 10E). Furthermore, the high-TMB/low-CIDGS group received the most benefits in terms of OS (*p* < 0.001; Figure 10E). The results described above indicated that CIDGS showed potential as a promising prognostic biomarker for HNSCCs.

### Predictive value of drug response and treatment agents targeting CIDGS

**FIGURE 11:**
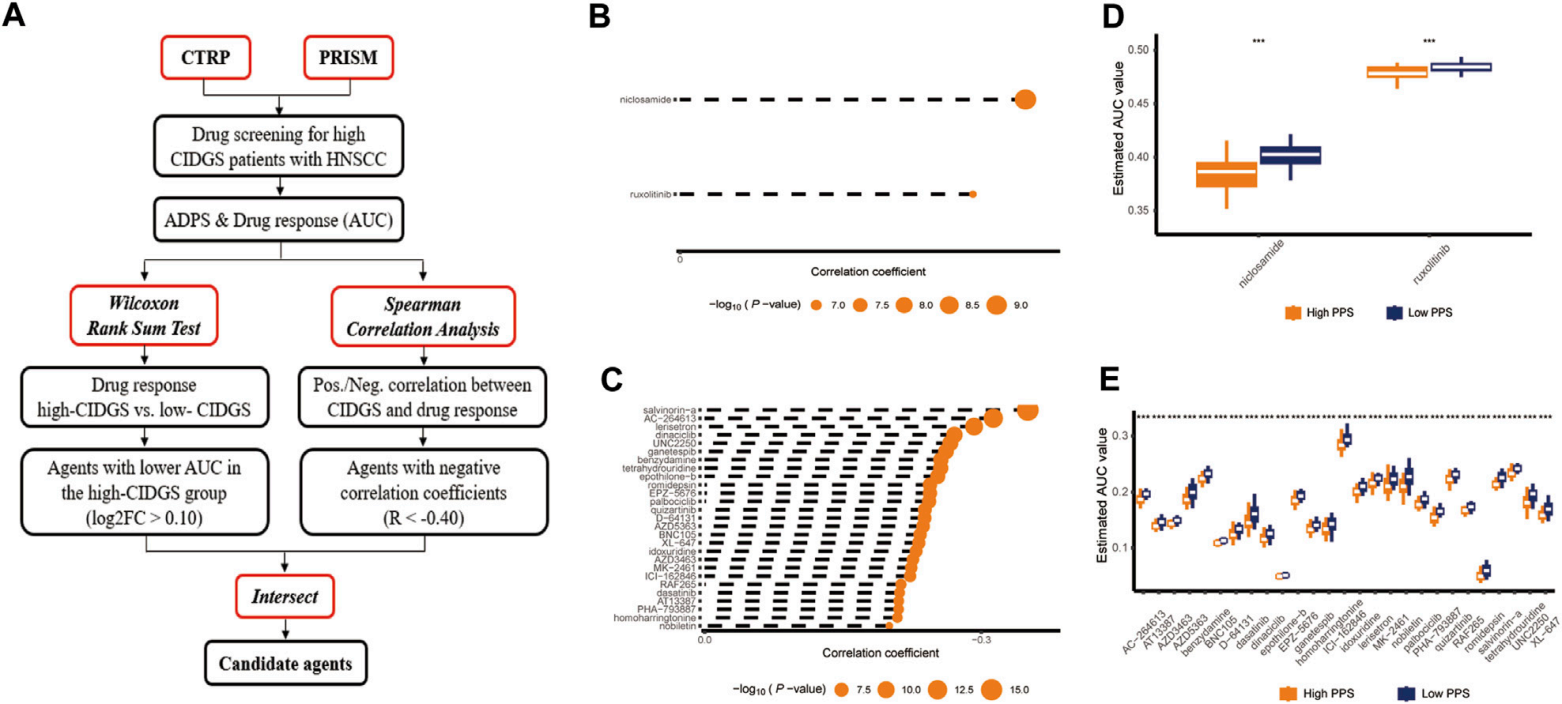
Identification of candidate drug with high sensitivity. **(A)** Schematic diagram illustrating the candidate drug with high sensitivity in patients with high CIDGS. **(B,C)** Correlations between CTRP- and PRISM-derived compounds. **(D,E)** Treatment response analysis of different agents. Lower *y*-axis values represent stronger drug sensitivity effects. ***p < 0.001.

**FIGURE 12:**
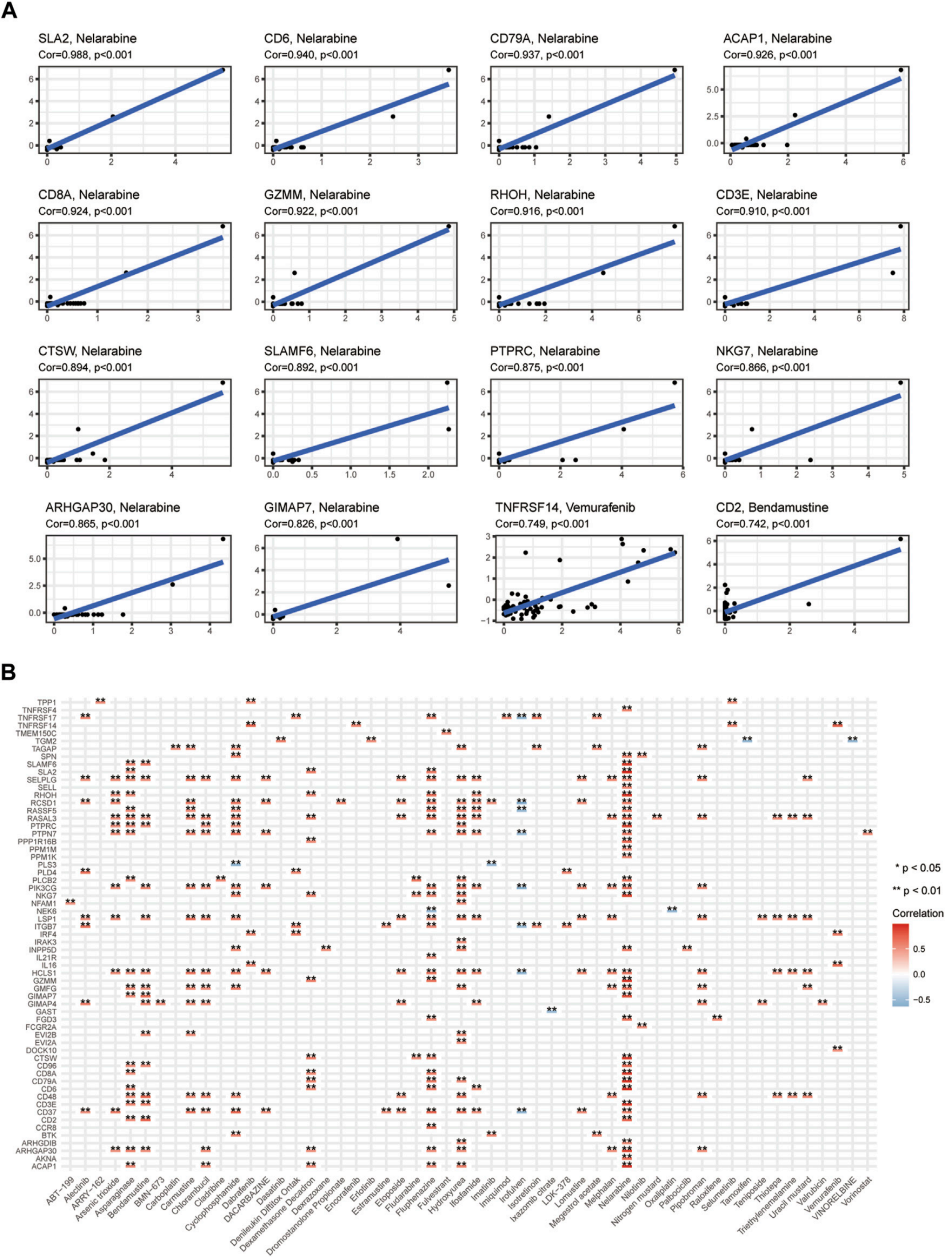
Potential drugs targeting CIDGS. **(A,B)** Correlations between CIDGS-related genes and therapeutic agents. *p < 0.05; **p < 0.01.

The results having indicated that the high-CIDGS group was more resistant to immunotherapy, we explored whether patients with high CIDGS scores responded to common antitumor agents (Figure 11A). Two CTRP-derived agents (niclosamide and ruxolitinib) and 27 PRISM-derived agents, including salvinorin-a, AC-264613, lerisetron, dinaciclib, and UNC2250, were identified. The estimated AUC values of antitumor agents showed a significantly negative relationship with CIDGS scores, with patients with low CIDGS scores showing significantly higher AUC values (Figure 11B–E). Furthermore, the relationship between CIDGS-related gene expression and drug sensitivity was assessed using Pearson’s correlation analysis (Figure 12). For example, increased *SLA2* expression was remarkably correlated with increased resistance to nelarabine (Cor = 0.988, *p* < 0.001; Figure 12A). Overall, the above-described results indicated that the CIDGS can predict the response to chemotherapy or small-molecule inhibitors in HNSCCs.

## Discussion

The unique heterogeneous features of HNSCCs have rendered available diagnostic and prognostic methods mostly ineffective, leading in many cases to misdiagnoses, undertreatment, or overtreatment (Salazar and Tabernero, 2014). Although advances in molecular techniques have enabled the molecular landscapes and subgroups of HNSCC to be identified (Walter et al., 2013; Keck et al., 2015; Wichmann et al., 2015), differences between sequencing platforms and analytical process as well as various prognostic relationships linked to subgroups continue to hamper their clinical application. Notably, treatment approaches aimed at HNSCC, such as the use of small molecule inhibitors (e.g., cetuximab) and ICIs (e.g., pembrolizumab, nivolumab), have become diversified (Ferris et al., 2016; Burtness et al., 2019). However, reliable biomarkers capable of identifying “high-risk” patients with HNSCC who stand to benefit from targeted therapies are urgently needed. Hence, more personalized assessment approaches that may help implement more efficient clinical decisions are needed. Herein, we used multilevel bioinformatics and machine learning algorithms to evaluate the association between immune-related genes and prognoses, as well as treatment benefits.

We used weighted correlation network analysis combined with consensus clustering algorithms as well as ssGSEA to detect immune-related genes in HNSCC. An integrative procedure involving the gene expression matrix was performed to identify the CIDGS. Ten machine learning algorithms were fitted to the training cohort based on the Leave-One-Out Cross-Validation framework. A combination of stepwise Cox regression (direction = both) and RSF was confirmed as the optimal model in two independent cohorts. Based on 85 algorithm combinations, the above integrative procedures were found to be the best fit for a model aimed at assessing the prognoses for HNSCCs, following which the dimensionality of variable algorithm combinations was further reduced, thereby simplifying as well as optimizing the model (Liu et al., 2022b). Kaplan–Meier and prognostic meta-analyses indicated that the CIDGS was a deleterious indicator of OS. Moreover, ROC and *C*-index analyses demonstrated that the CIDGS performed outstandingly in all assessed cohorts, indicating its potential for clinical application.

The tumor-node-metastasis grading system is widely applied to assess clinical outcomes and define treatment. Additionally, emerging biomarkers are significantly associated with clinical approaches and outcomes. Notably, the CIDGS functioned independently and its high performance in predicting the prognosis of HNSCCs based on *C*-index analysis was remarkable. The performance of 51 previously reported signatures of various functional gene combinations were retrieved to compare with that of the CIDGS. Although a few of these signatures have been actually utilized in clinical practice or validated in clinical trials (Magnes et al., 2021), *C*-index and ROC analyses suggested that the CIDGS was a better predictor than any of the signatures that have been already reported. The CIDGS, which was dimensionally reduced by incorporating 85 algorithm combinations displayed outstanding predictive capability, whereas the other signatures showed poor generalizability of the model due to overfitting. Moreover, a nomogram with accurate calibration curves further confirmed the clinical potential of CIDGS, indicating that the CIDGS showed potential as a promising surrogate model for assessing the prognosis of HNSCCs.

The results of multiple algorithms indicated that the low-CIDGS group showed high levels of infiltration by immune cells, including neutrophils, B cells, natural killer cells, and CD8^+^ T cells. Such an increase in the numbers of immune cells enhance antitumor immunity (Wu et al., 2013) thereby leading to better immunotherapeutic outcomes in patients with HNSCC showing a low CIDGS. Substantiating these observations, patients with a low CIDGS exhibit high expression levels of immune checkpoint associated genes, such as *BTLA*, *CD27*, *CTLA-4*, and *TIGIT*. Additionally, submap analysis of additional patient cohorts (IMvigor210 and GSE78220) was conducted to further evaluate the performance of the CIDGS and the molecular mechanisms underlying its efficacy. The low-CIDGS group was associated with immune-related pathways, such as natural killer cell-mediated cytotoxicity, transporters, and T cell receptor signaling pathways, whereas the high-CIDGS group was associated with biosynthesis-related pathways (*e.g.*, glycosaminoglycan, glycan) and biosynthesis and metabolism-related pathways (*e.g.*, amino sugar, fructose and mannose, nucleotide sugar, and galactose metabolism), which may explain the worse prognosis shown by patients with high CIDGS. Moreover, CIDGS-related genes were significantly associated with several pathways including apoptosis, platelet activation, natural killer cell-mediated cytotoxicity, and phagosome pathways. These results indicated that the CIDGS is associated with the carcinogenesis of HNSCC and thus may help identify immunotherapy-sensitive patients.

Cancer immunotherapy has remarkably improved the outcomes of patients with solid tumors, including a subset of patients with HNSCC. Two immune checkpoint inhibitors (nivolumab and pembrolizumab) are known to be highly beneficial to patients with unresectable, recurrent, or metastatic HNSCCs (Ferris et al., 2016; Burtness et al., 2019). In this study, the low-CIDGS group showed a lower TMB. Some genetic mutations generate clonal neoantigens which enhance neoantigen intratumor heterogeneity, thereby attenuating the antitumor response to ICI treatment (McGranahan et al., 2016). Patients with low CIDGS also display immunosuppressive (“immune-hot”) tumors characterized by high expression levels of immune cells as well as proteins, such as CTLA4 and PD-1. Notably, patients with low TMB and CIDGS experienced the highest OS. These results suggest that a low CIDGS leads to more backup lymphocyte resources and increased sensitivity to immunotherapy. With respect to the lower sensitivity shown to immunotherapy by the high-CIDGS group, specific drugs that could be beneficial for patients in this group were identified using two databases (Rahman et al., 2019; Luna et al., 2021) and comprehensive algorithms. Notably, several agents, such as niclosamide, ruxolitinib salvinorin-a, AC-264613, lerisetron, dinaciclib, and UNC2250, that may potentially induce positive antitumor responses in patients with high CIDGS were identified. These findings indicated that the CIDGS may be considered as an outstanding model that can be used to evaluate treatment outcomes of immunotherapy, with a high-CIDGS indicating resistance to immunotherapy.

The clinical performance of the CIDGS in HNSCC, although remarkable, was affected by certain limitations. This study was of a retrospective nature. Thus, a large-scale, prospective, multicenter study aimed at validating the clinical value of the CIDGS is needed. Moreover, the role of most CIDGS-related genes in HNSCCs should be further validated via both *in vivo* and *in vitro* experiments. Furthermore, some public datasets lack certain clinical and molecular features, thereby limiting any evaluation of a potential association between the CIDGS and some key variables. Thus, additional immunotherapy clinical trials aimed at validating the efficacy of the CIDGS for predicting the response of patients with HNSCC to immunotherapy are warranted.

## Conclusion

Using 85 algorithm combinations, we found a promising immune-related gene signature that may be utilized to evaluate the prognosis for HNSCC as well as the benefits conferred by immunotherapy and other therapeutic agents on patients with HNSCC. The findings of the current study indicate that the CIDGS shows potential as an outstanding model which may help make clinical decisions and predict the benefits of treatments targeting HNSCCs.

## Data Availability

The datasets presented in this study can be found in online repositories. The names of the repository/repositories and accession number(s) can be found in the article/Supplementary Material.
