# Supplementary figures and images for: Machine learning-based identification of a consensus immune-derived gene signature to improve head and neck squamous cell carcinoma therapy and outcome
Source: Front Pharmacol. 2024 Apr 10;15:1341346. doi: 10.3389/fphar.2024.1341346 (PMC11044683; doi:10.3389/fphar.2024.1341346)

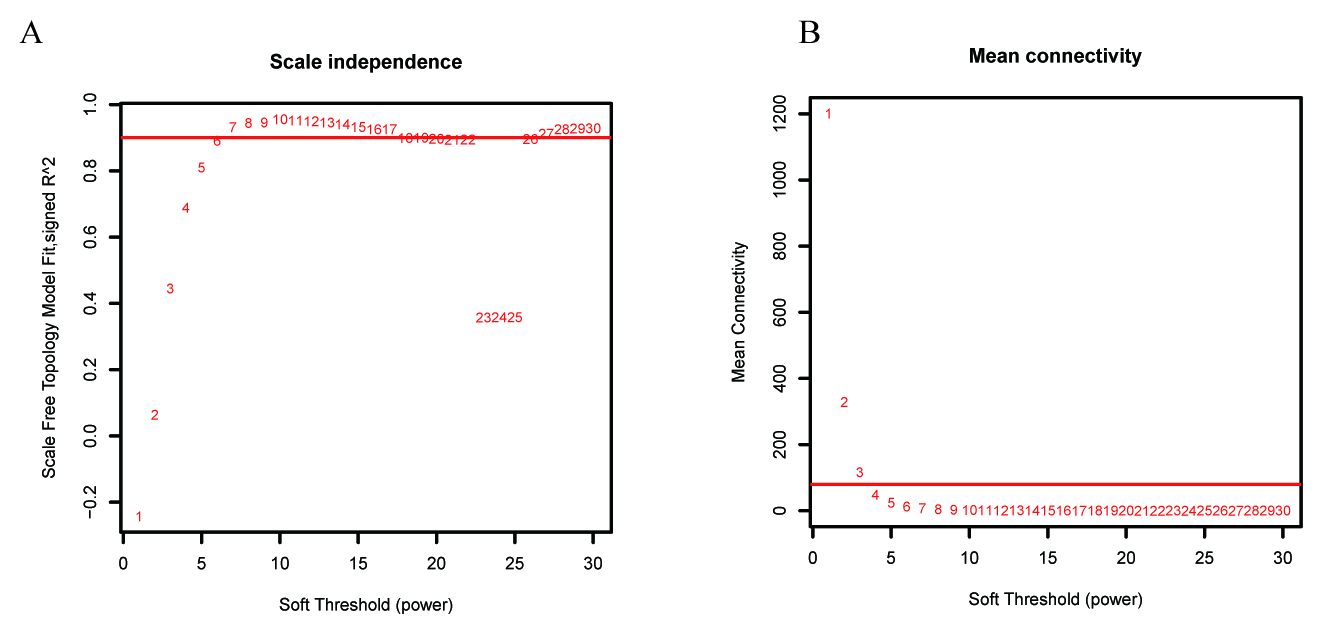

Supplement: Supplementary file 6 [file Image2.TIF]

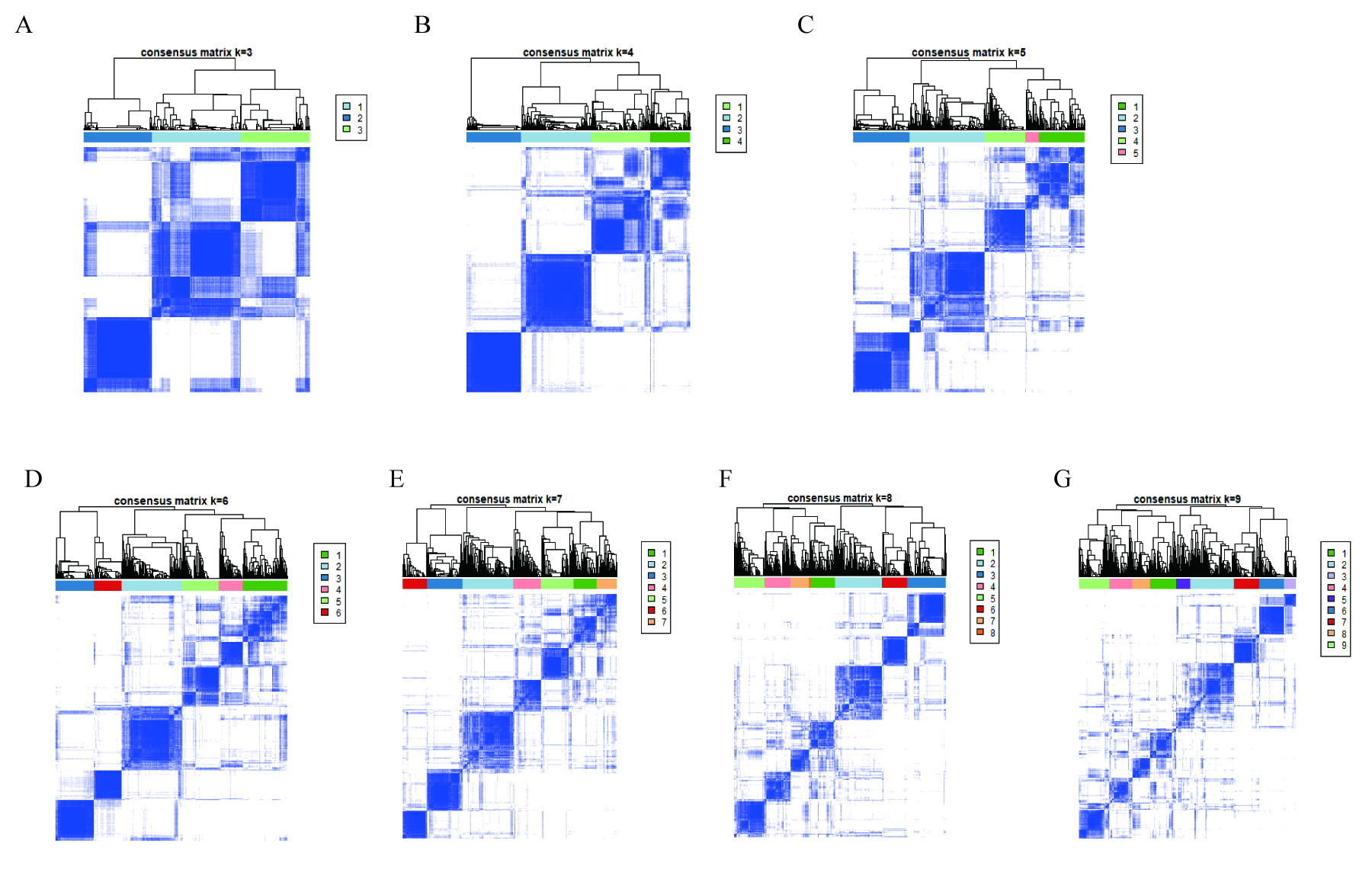

Supplement: Supplementary file 7 [file Image1.TIF]
